# Polyphenolic-Protein-Polysaccharide Complexes from *Hovenia dulcis*: Insights into Extraction Methods on Their Physicochemical Properties and In Vitro Bioactivities

**DOI:** 10.3390/foods9040456

**Published:** 2020-04-08

**Authors:** Ding-Tao Wu, Wen Liu, Mei-Lin Xian, Gang Du, Xin Liu, Jing-Jing He, Ping Wang, Wen Qin, Li Zhao

**Affiliations:** 1 College of Food Science, Sichuan Agricultural University, Ya’an 625014, Sichuan, China; DT_Wu@sicau.edu.cn (D.-T.W.); wlsicau@163.com (W.L.); sc_xianmeilin@163.com (M.-L.X.); liuxin1123712@163.com (X.L.); hjjxknl@163.com (J.-J.H.); wp234sicau@163.com (P.W.); qinwen@sicau.edu.cn (W.Q.); 2 Sichuan Provincial Institute for Food and Drug Control, Chengdu 611731, China; gd_fdc@163.com

**Keywords:** polyphenolic-protein-polysaccharide, *Hovenia dulcis*, extraction method, physicochemical properties, in vitro bioactivity

## Abstract

Seven extraction methods, including hot water extraction (HWE), pressurized water extraction (PWE), ultrasound-assisted extraction, microwave-assisted extraction, ultrasound-assisted enzymatic extraction, high-speed shearing homogenization extraction, and ultrasound-microwave-assisted extraction, were utilized to extract polyphenolic-protein-polysaccharide complexes (PPPs) from *Hovenia dulcis*. Next, their physicochemical properties and in vitro antioxidant activities, antiglycation effects, and inhibition activities on α-glucosidase and α-amylase were studied and compared. The findings from this study indicate that various extraction processes exhibit notable influences on the physicochemical properties and in vitro bioactivities of PPPs. Extraction yields, contents of polyphenolics and flavonoids, apparent viscosities, molecular weights, molar ratios of monosaccharide compositions, and ratios of amino acid compositions in PPPs varied in different extraction methods. Furthermore, 13 phenolic compounds in PPPs, including rutin, myricitrin, myricetin, quercetin, kaempferol, protocatechuic acid, gallocatechin, p-hydroxybenzoic acid, ampelopsin, quercetin-7,4′-diglucoside, dihydroquercetin, 5-methylmyricetin, and naringenin, were identified. The relatively strong in vitro antioxidant activities, antiglycation effects, and inhibition activities on α-glucosidase and α-amylase were determined in both PPP-W and PPP-P obtained by HWE and PWE, respectively. The high content of total polyphenolics may be one of the main contributors to their in vitro bioactivities. The findings have shown that the PWE method can be an appropriate method to prepare PPPs with strong bioactivities for application in the functional food industry.

## 1. Introduction

The polyphenolic-protein-polysaccharide complex (PPP) is a kind of natural extract that is rich in polyphenolic, protein, and polysaccharide obtained from medicinal and edible plants. Generally, PPPs possess remarkable antioxidant [1], antifungal [1], antibacterial [1], and antitussive activities [2,3]. For instance, PPPs extracted from *Cystoseira barbata* Tunisian seaweed possess the possibility to be developed as antioxidants and antibacterials [1]. Moreover, PPPs extracted from *Solidago canadensis* L. can be used as antitussives [2], and PPPs extracted from *Lythrum salicaria* can be used as bronchodilators [3]. Hence, the seeking and characterization of PPPs from medicinal and edible plants are important and necessary for the development of medicine and health foods.

*Hovenia dulcis* Thunb. (Rhamnaceae) is called “Guai Zao” or “Zhi Ju” in China [4]. It is a delicious and popular fruit, which is commonly utilized as a functional food and in folk medicine in China, Korea, and Japan [5]. Generally, *H. dulcis* can be utilized for the treatment of different diseases. It can promote digestion, produce saliva, quench thirst, antifebrile, and relieve hangovers [6]. Previous results indicated that both polyphenolics and polysaccharides obtained from *H. dulcis* exhibit a variety of bioactivities, such as antioxidant activities [4,7,8,9,10], hypoglycemic activities [10,11], and immunostimulatory activity [12]. In addition, natural polyphenolic-protein-polysaccharide complexes (PPPs) extracted from the peduncle of *H. dulcis* have also been proved to exhibit strong in vitro antioxidant activity, anti-glycation activity, and anti-hyperglycemic effects [6]. Therefore, natural PPPs extracted from *H. dulcis* possess good potential applications in the functional food field.

Extraction technologies are significantly influential for the utilization of the natural polysaccharide and polyphenolic-protein-polysaccharide complex from medicinal and edible plants, which can affect their extraction yields, structures, and bioactivities [13,14,15]. The conventional hot water extraction (HWE) method is widely applied to extract polysaccharides and polyphenolic-protein-polysaccharide complexes. However, it always possesses some defects, including long extraction time, high extraction temperature, and low extraction efficiency [16]. At present, several new and green extraction techniques have been carried out to prepare polysaccharides and PPPs, such as pressurized water extraction (PWE) [17,18], ultrasound-assisted extraction (UAE) [16,19], ultrasound-assisted enzymatic extraction (UAEE) [20,21], ultrasound-microwave-assisted extraction (UMAE) [22,23], microwave-assisted extraction (MAE) [24,25], and high-speed shearing homogenization extraction (HSHE) [26,27]. Many studies have revealed that extraction techniques can influence the physicochemical structures and bioactivities of natural polysaccharides. For instance, the polysaccharides extracted by the MAE method exhibit stronger antioxidant abilities than those of other methods [19], the polysaccharides extracted by the PWE method possess better inhibition activities on α-glucosidase and α-amylase than those of other methods [17], and the molecular weight of polysaccharides obtained by the HWE process is higher than those of other methods [24]. Nevertheless, there is limited study on the influences of various extraction processes on the bioactivities and physicochemical properties of PPPs from natural resources [15]. It is necessary to further evaluate the influences of various green extraction techniques on the physicochemical properties and bioactivities of natural PPPs extracted from medicinal and edible plants [14], which is important to develop the application in the medicine and health food fields. Our previous studies have demonstrated that in vitro bioactivities and physicochemical properties of PPPs obtained from *Hovenia dulcis* were significantly affected by various drying processes [6]. However, it is uncertain whether the physicochemical properties and bioactivities of PPPs are also influenced by various extraction processes.

Therefore, the influences of seven extraction processes, including HWE, PWE, UAE, MAE, UAEE, HSHE, and UMAE, on the physicochemical properties and in vitro bioactivities of PPPs were systematically studied. The findings from the present study could offer scientific fundaments to select suitable extraction methods to prepare PPPs with strong bioactivities for applications in the health food and pharmaceutical fields.

## 2. Materials and Methods

### 2.1. Material and Chemicals

The ripe and fresh peduncles of *Hovenia dulcis* were obtained in November of 2018 in AnKang City, Shaanxi Province, China. According to the previous study [6], the optimal drying process (microwave drying at 600 W) was used to dry the sample, and the dried sample was ground to pass through a 60 mesh sieve, and stored at −20 °C.

Acarbose, 4-nitrophenyl β-D-glucopyranoside (pNPG), α-glucosidase (10 U/mg), α-amylase (1000 U/mg), soluble starch, pectinase (1.15 U/mg), cellulase (800 U/g), rutin, myricetin, gallocatechin, quercetin, kaempferol, 2,2′-azino-bis(3-ethylbenzthiazoline-6-sulphonic acid) (ABTS), vitamin C (*Vc*), 2,2-diphenyl-1-(2,4,6-trinitrophenyl) hydrazyl (DPPH), and butylated hydroxytoluene (BHT), were obtained from Sigma-Aldrich (St. Louis, MO, USA). Heat stable α-amylase (40 U/mg) and glucoamylase (100 U/mg) were obtained from Solarbio (Beijing, China). Other chemicals utilized were of analytical grade.

### 2.2. Extraction of Polyphenolic-Protein-Polysaccharide Complexes (PPPs) by Different Methods

#### 2.2.1. Hot Water Extraction

Hot water extraction of PPPs was carried out by the formerly reported method [6]. In brief, samples (10.0 g) were extracted by 100.0 mL of 80% (*v*/*v*) ethanol to remove small molecules. The extracted residue was dried at 45 °C. Next, 300.0 mL of deionized water was used to extract PPPs from the dried residue at 95 °C for 3 h. After centrifugation at 4000× *g* for 10 min, the supernatant was collected and the heat stable α-amylase (1.0 U/mL) was added into the supernatant for the removal of starch in the extract at 65 °C for 6 h. When the KI-I reagent test of the extract was negative, the enzymes were inactivated at 90 °C for 1 h, and the mixture was centrifuged at 4000× *g* for 10 min. Next, the pancreatin (1.0 U/mL) was further added into the supernatant for the removal of proteins at 40 °C for 8 h. The enzymes were also inactivated at 90 °C for 1 h, and the mixture was also centrifuged at 4000× *g* for 10 min. After removing starch and proteins in the crude extract, three volumes of 95% ethanol (*v*/*v*) were used for the precipitation of crude PPPs and for the removal of enzymatic digestions of starch and proteins at 4 °C overnight. After centrifugation at 4000× *g* for 20 min, the precipitations (PPPs) were obtained and dissolved again in water. Furthermore, an Amicon ultra centrifugal filter device (molar mass cutoff: 3.0 kDa, Millipore, Billerica, MA, USA) was used to remove the low molecular weight compounds in PPPs by centrifugation at 3500× *g* for 25 min, such as free phenolics, free amino acids, and glucose and oligosaccharides released from starch. This step was repeated three times in order to thoroughly remove small molecules in the PPPs. Lastly, PPPs extracted by HWE was freeze dried and named as PPP-W.

#### 2.2.2. Pressurized Water Extraction

PWE was also performed by the formerly reported method with minor modifications [17]. Briefly, samples (10.0 g) were extracted by 100.0 mL of 80% (*v*/*v*) ethanol to remove small molecules. Next, the crude PPPs were obtained with 300.0 mL of deionized water by using a laboratory-scale high-pressure reactor (LEC-300, Shanghai Laibei Scientific Instruments Co., Ltd., Shanghai, China) at 95 °C and 1.5 MPa for 30 min. Lastly, after the same treatments as mentioned in Section 2.2.1, the PPP-P extracted by PWE were obtained.

#### 2.2.3. Ultrasound-Assisted Extraction

UAE was also carried out by the formerly optimized method with minor modifications [19]. Briefly, samples (10.0 g) were extracted by 100.0 mL of 80% (*v*/*v*) ethanol to remove small molecules. Next, PPPs were obtained with 300.0 mL of deionized water by using an ultrasonic homogenizer (JY92-IIN, Ningbo Scientz Biotechnology Co., Ltd., Ningbo, China) at 450 W and room temperature for 12 min. Lastly, after the same treatments as mentioned in Section 2.2.1, the PPP-U extracted by UAE was obtained.

#### 2.2.4. Microwave-Assisted Extraction

MAE was performed by the previous optimized method with minor modifications [19]. Briefly, samples (10.0 g) were extracted by 100.0 mL of 80% (*v*/*v*) ethanol to remove small molecules. Next, PPPs were obtained with 300.0 mL of deionized water by using a microwave oven (MKJ-J1-3, Qingdao Makewave Microwave Applied Technology Co., Ltd., Shandong, China) at 450 W for 8.0 min. Lastly, after the same treatments as mentioned in Section 2.2.1, the PPP-M extracted by MAE was obtained.

#### 2.2.5. Ultrasound-Assisted Enzymatic Extraction

UAEE was carried out by the formerly optimized method with minor modifications [20]. In brief, samples (10.0 g) were extracted by 100.0 mL of 80% (*v*/*v*) ethanol to remove small molecules. Next, 300.0 mL of deionized water, 0.5 g cellulase (800 U/g), and 0.01 g heat stable α-amylase (40 U/mg) were mixed with extraction residue. The extraction process was performed by utilizing an ultrasonic homogenizer (JY92-IIN, Ningbo Scientz Biotechnology Co., Ltd., Ningbo, China) at 450 W and room temperature for 12 min. Lastly, after the same treatment as mentioned in Section 2.2.1, the PPP-UE extracted by UAEE was obtained.

#### 2.2.6. Ultrasound-Microwave-Assisted Extraction

UMAE was performed by a combination of UAE and MAE as described above. Briefly, samples (10.0 g) were extracted by 100.0 mL of 80% (*v*/*v*) ethanol to remove small molecules. Next, UAE was carried out as mentioned in Section 2.2.3. Furthermore, the process was further performed by MAE as mentioned in Section 2.2.4. Lastly, after the same treatments as mentioned in Section 2.2.1, the PPP-UM extracted by UMAE was obtained.

#### 2.2.7. High-Speed Shearing Homogenization Extraction

HSHE was performed by the formerly reported method with minor modifications [27]. Briefly, samples (10.0 g) were extracted by 100.0 mL of 80% (*v*/*v*) ethanol to remove small molecules. After that, 500.0 mL of deionized water was added to the residue and then the process was performed by a high-speed shearing homogenization (AD500S-H, ANGNI INSTRUMENTS Co., Ltd., Shanghai, China) at 7000 rpm and room temperature for 5 min. Finally, after the same treatment as mentioned in Section 2.2.1, the PPP-HSH extracted by HSHE was obtained.

### 2.3. Physicochemical Characterization of PPPs

#### 2.3.1. Analysis of Chemical Compositions

The proteins, total uronic acids, total polysaccharides, total polyphenolics, and total flavonoids in PPPs were measured by the colorimetric methods based on our formerly reported methods [28,29], respectively. Bovine serum albumin, galacturonic acid, glucose, gallic acid, and rutin were used as the reference substances, respectively.

#### 2.3.2. Determination of Molecular Weights, Apparent Viscosities, Monosaccharide Compositions, and Amino Acid Compositions

The molecular weights (*M_w_*) and polydispersities (*M_w_/M_n_*) of PPPs were determined by high-performance size-exclusion chromatography and a multi-angle laser light scattering and a refractive index detector (HPSEC-MALLS-RID, Wyatt Technology Co., Santa Barbara, CA, USA) [28]. The Shodex OHpak SB-806M HQ (300 mm × 8.0 mm, i.d.) column was utilized. For the determination of polysaccharide fraction, the *dn/dc* value of 0.15 mL/g was used. For the determination of polysaccharide-protein fractions, the *dn/dc* value of 0.164 mL/g was used. The apparent viscosities of PPPs were measured by using a Discovery Hybrid Rheometer-1 (DHR-1, TA Instruments, New Castle DE, USA) based on the formerly reported method [30]. Furthermore, monosaccharide compositions of PPPs were measured by high-performance liquid chromatography (HPLC, ThermoFisher scientific, Waltham, MA, USA) coupled with a ZORBAX Eclipse XDB-C18 column (4.6 × 250 mm i.d. 5 µm) and a diode array detector (DAD, ThermoFisher scientific, Waltham, MA, USA) according to the formerly reported method [30]. Moreover, amino acid compositions of PPPs were also measured by an automated amino acid analyzer (HITACHI L-8900, Amino Acid Analyzer, Japan) according to the formerly reported method [6].

#### 2.3.3. Fourier Transform Infrared (FT-IR) Spectroscopy Analysis

The FT-IR spectroscopy experiment of PPPs was carried out by a Nicolet iS 10 FT-IR (ThermoFisher scientific, Waltham, MA, USA) based on our formerly reported method [28].

#### 2.3.4. Identification of Phenolic Compounds

The polyphenolic compounds in PPPs were firstly released by the heat-stable a-amylase, glucoamylase, pectinase, and trypsin digestions, and the analysis was then carried out by utilizing an UPLC 1290 series system coupled with a high resolution quadrupole time-of-flight mass spectrometer (G6545 Q-TOF-MS, Agilent Technologies, Palo Alto, CA, USA) according to the previously reported methods [6]. A ZORBAX SB-C18 column (2.1 mm × 50 mm, 1.8 μm, Agilent Technologies, Palo Alto, CA, USA) was utilized for the separation of phenolic compounds released from PPPs.

### 2.4. Evaluation of In Vitro Bioactivities of PPPs

#### 2.4.1. In Vitro Antioxidant Activities

The ABTS, DPPH, and nitric oxide (NO) radical scavenging activities, and the ferric reducing antioxidant powers (FRAP) of PPPs were detected based on the formerly reported methods [16,17]. The positive control was Vitamin C or BHT. PPPs were detected at five different concentrations, which ranged from 0.05 to 0.50 mg/mL. Finally, a logarithmic regression curve was established to calculate IC_50_ values (mg/mL) of PPPs.

#### 2.4.2. In Vitro Antiglycation Activities

Antiglycation activities of PPPs were also detected based on the formerly reported method [6]. The positive control was Aminoguanidine (AG). PPPs were detected at different concentrations, which ranged from 0.25 to 2.00 mg/mL. A logarithmic regression curve was established to calculate IC_50_ values (mg/mL) of PPPs.

#### 2.4.3. In Vitro α-Amylase and α-Glucosidase Inhibitory Activities

The α-glucosidase and α-amylase inhibitory activities of PPPs were detected by the formerly reported methods [6]. The positive control was acarbose. PPPs were detected at five different concentrations, which ranged from 10 to 160 μg/mL. Finally, a logarithmic regression curve was established to calculate IC_50_ values (μg/mL) of PPPs.

### 2.5. Statistical Analysis

The data were expressed as means ± standard deviations. Statistical analysis was carried out by one-way analysis of variance (ANOVA), followed by Duncan’s test. Statistically significant was determined when values of *p* < 0.05.

## 3. Results and Discussions

### 3.1. Physicochemical Characteristics of PPPs

#### 3.1.1. Chemical Compositions

Table 1 shows the extraction yields and chemical compositions of PPPs. The results suggested that the extraction yields of PPPs were remarkably impacted by extraction methods, which ranged from 2.16% to 3.79%. The extraction yields were similar to the former results ranging from 2.61% to 5.20% [6]. Indeed, the extraction yields of PPP-W, PPP-P, PPP-U, and PPP-UE obtained by HWE, PWE, UAE, and UEAE, respectively, were higher than those of PPP-M, PPP-HSH, and PPP-UM, which might be due to the fact that the microwave and high-speed shearing homogenization could degrade the structures of PPPs [14]. Besides, the contents of polysaccharides in PPPs varied from 29.32% to 42.12%, which were similar to the former results [10]. The contents of proteins in PPPs varied from 18.44% to 26.75%. Both polysaccharides and proteins were important biological components in PPPs. Our results suggest that proteins in PPPs might be beneficial for the binding polyphenols according to glycosylation [31]. The contents of uronic acids in PPPs varied from 2.60% to 4.95%, which were similar to these studies [8,10]. Furthermore, the findings suggested that extraction processes also remarkably impacted the contents of polyphenolics and flavonoids in PPPs, which varied from 156.59 to 277.56 mg GAE/g, and from 80.09 to 141.10 mg RE/g, respectively. The existence of PPPs in *H. dulcis* was confirmed [1]. The contents of polyphenolics in PPP-W and PPP-P were remarkably higher than those in other tested PPPs, which might be correlated with this fact that polyphenolics can be associated with polysaccharides and/or proteins more easily because of high temperature and high pressure during HWE and PWE extraction [32], as well as the degradation of PPPs during ultrasound- and microwave-assisted extraction [15].

#### 3.1.2. Molecular Weights, Apparent Viscosities, and Monosaccharide Compositions

**Figure 1:**
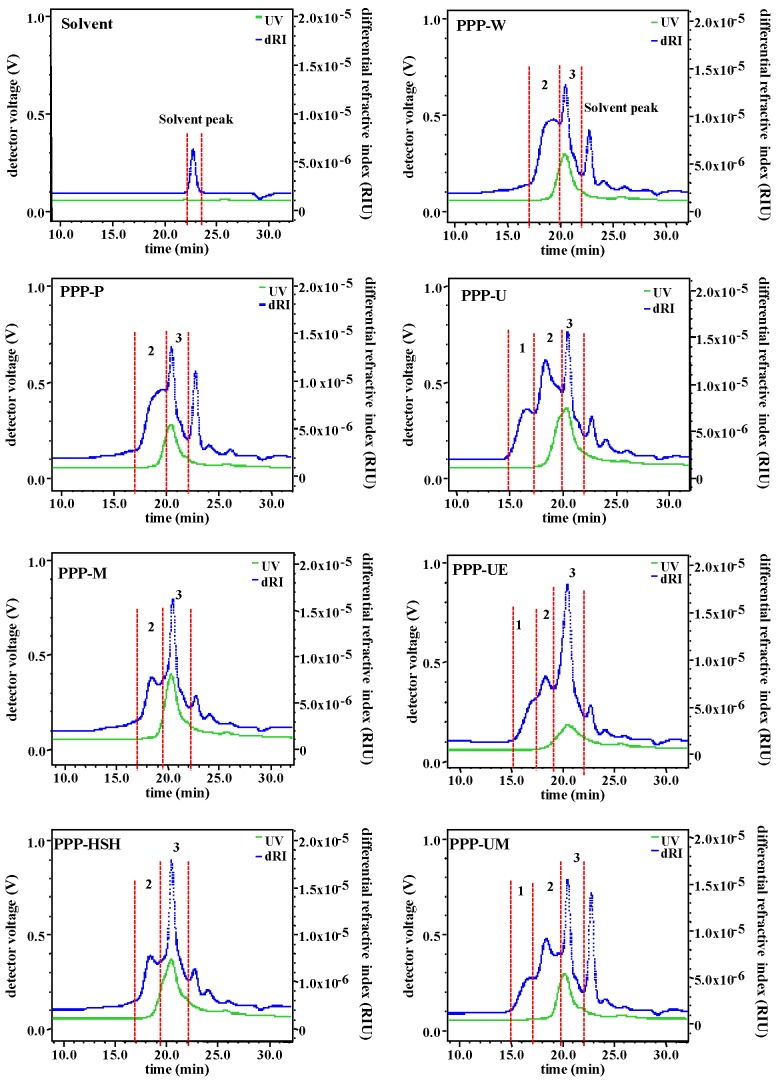
High performance size exclusion chromatograms of PPPs. The codes of samples were the same as in Table 1.

Extraction methods can influence molecular weights, viscosities, and monosaccharide compositions of polysaccharides, which can also affect the bioactivities of polysaccharides [17,33]. Accordingly, *M_w_*, viscosities, and monosaccharide compositions of PPPs extracted by seven extraction methods were studied. Figure 1 shows the HPSEC-RID-UV chromatograms of PPPs. The results indicated that molecular weight distributions of PPPs were influenced by various extraction processes significantly. Three fractions (fractions 1 to 3) have been found in PPP-U, PPP-UE, and PPP-UM, but only two fractions (fraction 2 and fraction 3) have been found in PPP-W, PPP-P, PPP-M, and PPP-HSH. Furthermore, the UV (280 nm) signals were found in fraction 2 and fraction 3, indicating that phenolic compounds and proteins might bond on polysaccharide fractions. Table 2 summarized the *M_w_* of fraction 2 and fraction 3 in PPP-W, PPP-P, PPP-M, and PPP-HSH, which varied from 4.905 × 10^4^ to 9.170 × 10^4^ Da and from 0.794 × 10^4^ to 1.763 × 10^4^ Da, respectively. The fraction 1 in PPP-U, PPP-UE, and PPP-UM might be correlated with the release of polysaccharides because of ultrasonic processes [15], and molecular weights of fractions 1 to 3 in PPP-U, PPP-UE, and PPP-UM varied from 2.768 × 10^5^ to 3.612 × 10^5^ Da, from 7.563 × 10^4^ to 9.113 × 10^4^ Da, and from 1.078 × 10^4^ to 1.865 × 10^4^ Da, respectively. Results indicated that the molecular weights of PPP-U, PPP-UE, and PPP-UM obtained by ultrasonic treatments were much higher than those of PPPs obtained by other methods. Moreover, the polydispersities of fraction 2 and fraction 3 in PPP-W, PPP-P, PPP-M, and PPP-HSH varied from 1.083 to 1.702 and from 1.074 to 1.138, respectively, which matched with the HPSEC-RID chromatograms. The polydispersities of fractions 1 to 3 in PPP-U, PPP-UE, PPP-UM varied from 1.155 to 1.161, from 1.076 to 1.349, and from 1.104 to 1.154, respectively, which also matched with the HPSEC-RID chromatograms.

**Figure 2:**
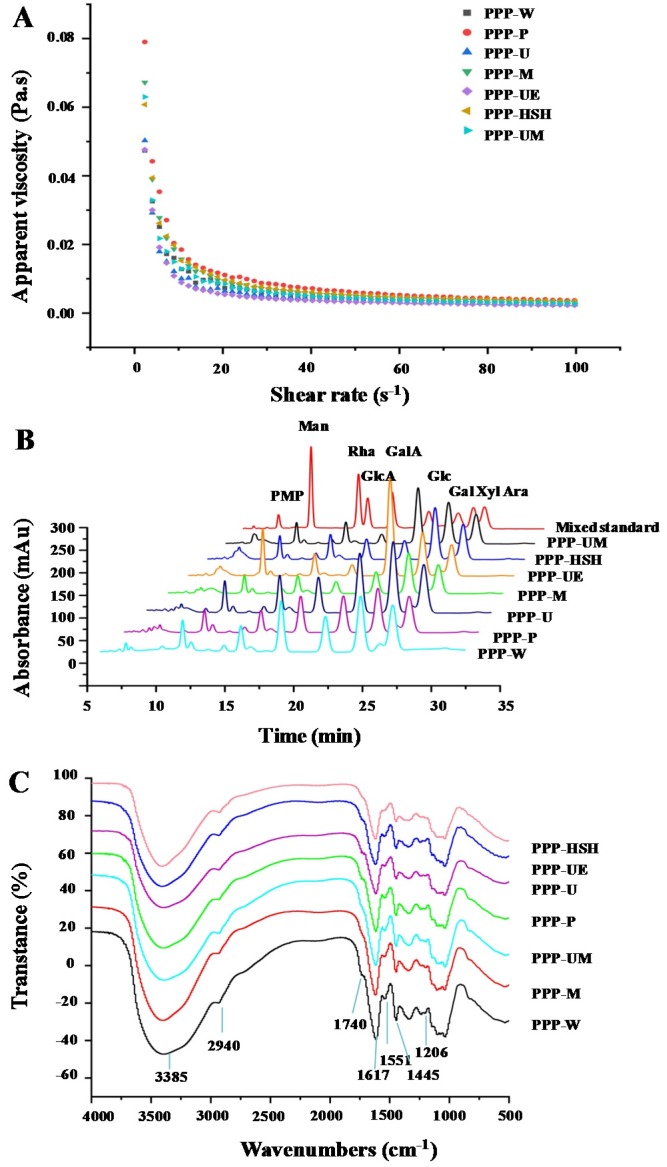
Dependences of apparent viscosities on the shear rate (**A**), high performance liquid chromatography profiles (**B**), and FT-IR spectra (**C**) of PPPs. The codes of samples were the same as in Table 1; PMP, 1-phenyl-3-methyl-5-pyrazolone, Man, mannose; Rha, rhamnose; GlcA, glucuronic acid; GalA, galacturonic acid; Glc, glucose; Gal, galactose; Xyl, xylose; Ara, arabinose.

The apparent viscosities of PPPs of 10.0 mg/mL at 25 °C were shown in Figure 2A. All PPPs exhibited shear-thinning behavior. PPPs exhibited non-Newtonian shear thinning behavior at a low shear rate range, but at a high shear rate range, all PPPs showed Newtonian flow behavior. Results were similar to the former findings [6]. The apparent viscosities of PPPs were affected by various extraction processes significantly. The highest apparent viscosity was found in PPP-P among all PPPs, while the lowest viscosity was found in PPP-UE. Commonly, the viscosity of polysaccharides is correlated with their chemical structures, such as degrees of esterification (Des), molecular weights, and molecular weight distributions [34], which may also influence the viscosity of the PPPs.

Moreover, studies have revealed that monosaccharide compositions of polysaccharides and polyphenolic-protein-polysaccharide complexes influence their bioactivities [6,19]. Figure 2B suggested that the HPLC-UV profiles of PPPs were similar, and the constituent monosaccharides of PPPs were determined as GalA, Gal, Man, Rha, GlcA, Glc, Xyl, and Ara, indicating that the types of constituent monosaccharides of PPPs were similar. Table 2 showed the molar ratios of constituent monosaccharides mentioned above, which were influenced by various extraction processes. Similar results also showed that extraction techniques influenced the molar ratios of monosaccharide compositions in the PPPs, but had no effects on their types [15]. In addition, the major monosaccharides in PPPs significantly varied by different extraction methods. Some portions of monosaccharide could be promoted by different extraction procedures [5]. These results showed that extraction techniques influenced the physicochemical structures of PPPs. The existence of pectic-polysaccharides in PPPs was also confirmed [28,35]. Results showed that rhamnogalacturonan I (RG I), homogalacturonan (HG), arabinogalactan (AG II), glucan, and glucomannan might be in PPPs according to their constituent monosaccharides [19,28].

#### 3.1.3. Amino Acid Compositions and Phenolic Compositions

Table 3 summarized that a total of 16 amino acids were found in the protein fractions of PPPs. Results showed that the types of amino acids in protein fractions of PPPs extracted by seven extraction methods were the same, but their ratios significantly varied between different extraction processes. In addition, the ratios of essential amino acids (EEA) in PPPs varied from 24.48% to 34.85%, which were similar to our study [6]. Arginine, aspartic acid, serine, glutamic acid, alanine, threonine, glycine, and leucine were determined to be the major amino acids in all PPPs, which varied from 5.89% to 11.76%, from 7.01% to 10.66%, from 6.80% to 8.91%, from 9.19% to 15.77%, from 8.62% to 11.20%, from 4.53% to 9.26%, from 4.27% to 12.10%, and from 5.06% to 7.90%, respectively. Similar trends were found in the PPPs obtained from *H. dulcis* [6] and *Cystoseira barbata* [1].

Furthermore, polyphenols can selectively and unselectively interact with polysaccharides and proteins, which may influence their stabilization and bioactivities [10]. The contents of polyphenolics in PPPs were studied, which varied between different extraction methods (Table 1). Individual phenolics in PPPs, which were released by the heat-stable a-amylase, glucoamylase, pectinase, and trypsin digestions and were further determined by utilizing UPLC-Q-TOF-MS. Results indicated that a total of 13 phenolics were found in PPPs (Table 4). The retention time, formula, molecular ion, and scores obtained by the Personal Compound Database Library Manager B. 08.00 software and the Traditional Chinese Medicine Database (Agilent Technologies, Palo Alto, CA, USA) of each compound were showed in Table 4. The 13 phenolics were rutin, myricitrin, myricetin, quercetin, kaempferol, protocatechuic acid, gallocatechin, p-hydroxybenzoic acid, ampelopsin, quercetin-7,4′-diglucoside, dihydroquercetin, 5-methylmyricetin, and naringenin. Similar results were also found in the previous study [6]. The phenolics might be spontaneously bonded with polysaccharides and/or proteins by the ionic interaction, the hydrogen bonding, and the hydrophobic interaction [6].

#### 3.1.4. FT-IR Spectra

Chemical structures of PPPs were measured according to the FT-IR spectra. Figure 2C showed that the FT-IR spectra of PPPs obtained by seven extraction methods were similar, suggesting that chemical structures of PPPs were similar. Briefly, the absorptions at 3385 cm^−1^ and 2940 cm^−1^ represent the absorptions of O-H and C-H, respectively [35]. The band at 1740 cm^−1^ represents the esterified carboxylic groups [24]. Besides, the band at 1617 cm^−^^1^ represents the C = O, indicating that uronic acids exist in PPPs [10,17]. The bands at 1617 cm^−^^1^ and 1551 cm^−^^1^ are amide I and amide II regions, indicating that proteins were bonded with polysaccharides in PPPs [1]. The band at 1445 cm^−^^1^ represents C-H or O-H [28]. Furthermore, the phenyl-OH structure was confirmed by the peak at about 1206 cm^−^^1^ [14,36], indicating the existence of polyphenolics (Table 1 and Table 4). Moreover, the analysis was also applied to detect the degrees of esterification (Des) of PPPs. Table 1 showed that the Des of PPPs were impacted by various extraction processes, which ranged from 1.03% to 6.80%.

### 3.2. Impacts of Extraction Methods on the In Vitro Bioactivities of PPPs

#### 3.2.1. In Vitro Antioxidant Activities

**Figure 3:**
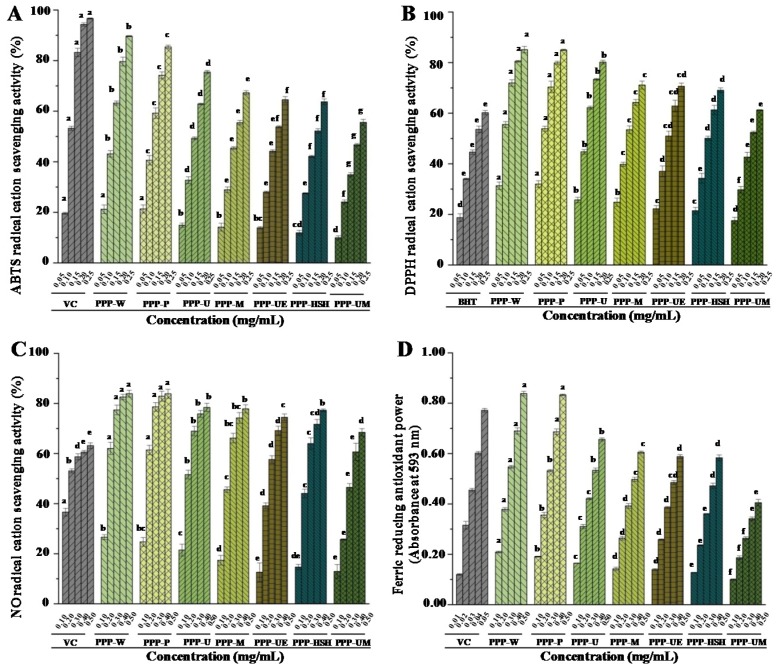
ABTS (**A**), DPPH (**B**), and nitric oxide (**C**) radical scavenging activities, as well as ferric reducing antioxidant powers (**D**) of PPPs. The codes of samples were the same as in Table 1; ABTS, 2,2′-azino-bis(3-ethylbenzthiazoline-6-sulphonic acid); DPPH, 2,2-diphenyl-1-(2,4,6-trinitrophenyl) hydrazyl; *Vc*, vitamin C; BHT, butylated hydroxytoluene; The error bars are standard deviations; Significant (*p* < 0.05) differences are shown by data bearing different letters (a–h); Statistical significances were performed by ANOVA and Duncan’s test.

The previous study has revealed that PPPs exhibit strong in vitro antioxidant effects [6]. Therefore, the impact of extraction processes on the antioxidant effects of PPPs was studied. Figure 3 displayed the antioxidant effects of PPPs extracted by various extraction processes, including ABTS, DPPH, and NO radical scavenging activities, as well as ferric reducing antioxidant powers (FRAP). The findings showed that antioxidant effects of PPPs obtained by seven extraction processes exhibited dose-dependent manners (Figure 3). All PPPs exerted strong ABTS, DPPH, and NO radical scavenging activities when compared with positive controls. The IC_50_ values of ABTS, DPPH, and NO radical scavenging activities of PPPs, extracted by different extraction methods, varied from 0.11 mg/mL to 0.22 mg/mL, from 0.08 mg/mL to 0.18 mg/mL, and from 0.16 mg/mL to 0.33 mg/mL, respectively. Moreover, all PPPs also exhibited remarkable ferric-reducing antioxidant powers when compared with the positive control (Figure 3D). The findings indicate that antioxidant effects of PPPs were remarkably impacted by various extraction processes. The highest antioxidant activities were found in both PPP-W and PPP-P among all PPPs, and the lowest antioxidant activities were found in PPP-UM. Commonly, the antioxidant effects of PPPs are related to the physicochemical compositions (contents of polyphenolics, total proteins, and uronic acids), compositional monosaccharides, molecular weights, and degrees of esterification [1,14,28,37,38,39]. Therefore, the higher antioxidant activities determined in both PPP-W and PPP-P might be correlated with the higher contents of polyphenolics, proteins, and uronic acids, and lower molecular weights (Table 1 and Table 2) when compared with other tested PPPs. Indeed, the antioxidant abilities of PPPs were stronger than that of polysaccharides obtained from *Hovenia dulcis* [8], and were positively related to their contents of polyphenolics (Table 1 and Figure 3), which further confirmed that the bonded polyphenolics could be one of the main contributors to the antioxidant abilities of PPPs [19,40]. Indeed, some phenolic compounds in PPPs determined by UPLC-Q-TOF-MS possess obvious in vitro antioxidant activities, such as gallocatechin [41], kaempferol [42], myricetin [43], and quercetin [44]. Previous study has also revealed that the main mechanisms for the antioxidant activities of polyphenols might be the metals chelation, the H-atom transfer, and the electron transfer [45]. Furthermore, the antioxidant activities of PPP-W and PPP-P were much stronger than that of other natural complexes, such as polysaccharide-protein complex from black soybean [46] and glycoproteins from *Ganoderma atrum* [47]. Finally, in consideration of the extraction efficiency, the results indicated that PWE could be a potential extraction method to prepare PPPs with strong antioxidant abilities for industrial applications.

#### 3.2.2. In Vitro Antiglycation Activities

**Figure 4:**
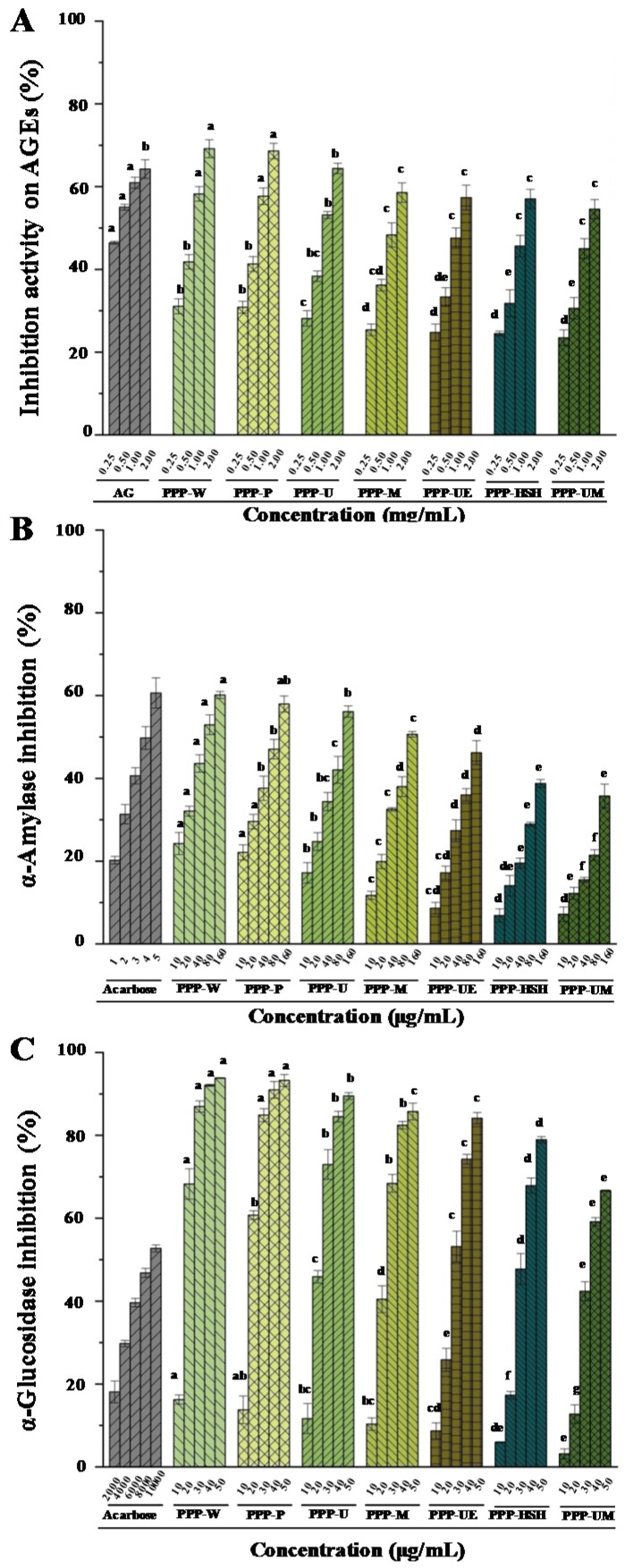
Inhibition activities on AGEs (**A**), and inhibitory activities on α-amylase (**B**) and α-glucosidase (**C**) of PPPs. The codes of samples were the same as in Table 1; AG, aminoguanidine; AGEs, advanced glycation end products; The error bars are standard deviations; Significant (*p* < 0.05) differences are shown by data bearing different letters (a–h); Statistical significances were performed by ANOVA and Duncan’s test.

Commonly, the advanced glycation end products (AGEs) could bring about aging, diabetic complications, and arteriosclerosis [48]. The mechanism of antiglycation might be associated with the antioxidant effects. The previous study has revealed that PPPs exhibit remarkable antiglycation effects [6]. Therefore, the antiglycation activities of PPPs extracted by seven extraction methods were investigated. Figure 4A indicated that both PPPs and the positive control (AG) showed remarkable antiglycation activities. The IC_50_ values of inhibition effects on AGEs of PPPs ranged from 0.71 mg/mL to 1.49 mg/mL. Moreover, in comparison with the positive control (IC_50_ = 0.33 mg/mL), PPPs possessed good antiglycation activities. Furthermore, results demonstrated that antiglycation effects of PPPs were remarkably affected by various extraction processes. The inhibition abilities on the formation of AGEs of PPPs were in the order of PPP-W/PPP-P > PPP-U > PPP-M > PPP-UE > PPP-HSH > PPP-UM, which positively correlated with the antioxidant activities of PPPs. In addition, the antiglycation activities of PPPs were higher than those of polysaccharides extracted from *Actinidia arguta* [49].

It is well known that the mechanism of glycation is one of reducing sugar and protein in a spontaneous reaction, which is known as a non-enzymatic amino-carbonyl reaction, bringing about the formation of AGEs [49]. The study has indicated that the antioxidant and the radical scavenger could restrain the process and inhibit the formation of AGEs [49]. Therefore, PPPs can inhibit the formation of AGEs by exerting free radical scavenging activities. Obviously, the fact that PPPs exerted remarkable antiglycation effects might be correlated with the strong antioxidant effects, which might be also related to total flavonoids and total polyphenolics. Furthermore, flavonoids and phenolic compounds can inhibit AGEs formation through the strong antioxidant properties [50]. Therefore, the higher antiglycation activities determined in both PPP-W and PPP-P might be associated with their higher antioxidant effects, and higher contents of total flavonoids and polyphenolics. Finally, results also indicated that the PWE method could be an effective one to prepare PPP-P with remarkable antiglycation activities for industrial applications.

#### 3.2.3. In Vitro Inhibitory Activities on α-Amylase and α-Glucosidase

Previous study has demonstrated that an important method to treat type 2 diabetes is to inhibit the activities of α-amylase and α-glucosidase [17]. The former study revealed that PPPs possessed strong α-amylase inhibitive abilities and α-glucosidase inhibitive abilities [6]. Therefore, influences of extraction procedures on enzyme inhibition activities of PPPs were studied. Figure 4B,C indicated that PPPs possessed remarkable α-amylase and α-glucosidase inhibitions in dose-dependent manners. The IC_50_ values of α-amylase inhibition and α-glucosidase inhibition of PPPs ranged from 69.98 ± 1.30 to 509.62 ± 10.07 μg/mL, and from 16.10 ± 0.65 to 36.23 ± 0.75 μg/mL, respectively. Results indicated that extraction methods were important to the enzyme inhibition activities of PPPs. Both PPP-W and PPP-P possessed stronger α-glucosidase and α-amylase inhibitive abilities than those of other tested PPPs. The lowest α-glucosidase and α-amylase inhibitive abilities were also determined in PPP-UM. Moreover, in comparison with the positive control, all PPPs exhibited strong α-glucosidase inhibitive effects and moderate α-amylase inhibitive effects. However, compared with polysaccharides obtained from okra [51], *Momordica charantia* [37], and green tea [52], both PPP-W and PPP-P exhibited higher α-amylase inhibition activities. Furthermore, the α-amylase and α-glucosidase inhibitory activities of PPPs were higher than those of polysaccharides obtained from *Hovenia dulcis* [10], but lower than those of flavonoids obtained from *Hovenia dulcis* [11], and the α-glucosidase and α-amylase inhibitive effects were also positively correlated with the contents of polysaccharides and flavonoids. Therefore, flavonoids might also play an important role in α-glucosidase and α-amylase inhibitive abilities [11,35,53]. In addition, the total flavonoids in *Hovenia* were competitive inhibitors for α-amylase and non-competitive inhibitors for α-glucosidase [11], respectively. Finally, results showed that the PWE method could be used to prepare PPPs with remarkable α-amylase and α-glucosidase inhibitive abilities.

## 4. Conclusions

Noticeable influences caused by different extraction methods on the physicochemical properties and in vitro bioactivities of PPPs have firstly been compared in this study. The highest contents of total polyphenolics and flavonoids were determined in both PPP-W and PPP-P among all PPPs. Furthermore, the stronger antioxidant abilities, antiglycation effects, and inhibition activities on α-glucosidase and α-amylase were determined in PPP-W and PPP-P among all PPPs. Results suggested that the high contents of polyphenolics and flavonoids in PPPs could be one of the major contributors to their high in vitro bioactivities. The findings from this study could offer scientific fundaments to select suitable extraction methods to prepare PPPs, and PWE methods could be an efficient extraction method to obtain PPPs with relatively strong bioactivities for applications in the pharmaceutical and functional food industries.

## Figures and Tables

**Table 1:** Chemical compositions of PPPs obtained by seven extraction methods.

Chemical Compositions	PPPs Extracted from the Peduncles of *H. dulcis*						
	PPP-W	PPP-P	PPP-U	PPP-UE	PPP-UM	PPP-M	PPP-HSH
Extraction yields (%)	3.52 ± 0.28 ^a,b^	3.79 ± 0.22 ^a^	3.37 ± 0.15 ^b^	3.50 ± 0.30 ^a,b^	3.13 ± 0.18 ^b^	2.16 ± 0.20 ^c^	2.50 ± 0.16 ^c^
Total polysaccharides (%)	33.34 ± 0.53 ^b^	40.23 ± 1.09 ^a^	42.12 ± 1.56 ^a^	40.88 ± 1.01 ^a^	29.32 ± 1.08 ^c^	29.49 ± 1.76 ^c^	30.43 ± 1.32 ^c^
Total uronic acids (%)	4.95 ± 0.61 ^a^	4.02 ± 0.98 ^ab^	4.07 ± 0.69 ^a,b^	2.83 ± 0.69 ^bc^	2.91 ± 0.78 ^b,c^	2.60 ± 0.33 ^c^	3.08 ± 0.47 ^b,c^
Total proteins (%)	26.75 ± 0.56 ^a^	25.45 ± 0.94 ^b^	18.44 ± 0.23 ^f^	24.36 ± 0.36 ^c^	19.70 ± 0.49 ^e^	21.53 ± 0.64 ^d^	25.73 ± 0.82 ^a,b^
Degrees of esterification (%)	6.43 ± 0.18 ^b^	6.80 ± 0.21 ^a^	4.55 ± 0.15 ^c^	4.29 ± 0.20 ^c^	4.55 ± 0.22 ^c^	1.03 ± 0.05 ^e^	3.74 ± 0.10 ^d^
TPC (mg GAE/g)	277.56 ± 1.80 ^a^ (27.76%)	273.52 ± 2.40 ^a^ (27.35%)	226.92 ± 3.93 ^b^ (22.69%)	189.73 ± 4.25 ^d^ (18.97%)	156.59 ± 2.44 ^f^ (15.66%)	199.03 ± 3.29 ^c^ (19.90%)	179.71 ± 7.58 ^e^ (17.97%)
TFC (mg RE/g)	141.10 ± 5.64 ^a^ (14.11%)	139.57 ± 4.18 ^a^ (13.96%)	112.69 ± 2.23 ^b^ (11.27%)	96.71 ± 3.29 ^c^ (9.67%)	80.09 ± 2.08 ^d^ (8.01%)	97.30 ± 1.39 ^c^ (9.73%)	85.63 ± 1.77 ^d^ (8.56%)

PPP-W, PPP-P, PPP-U, PPP-UE, PPP-UM, PPP-M, and PPP-HSH, polyphenolic-protein-polysaccharide complexes extracted by hot water extraction (HWE), pressurized water extraction (PWE), ultrasound-assisted extraction (UAE), ultrasound-assisted enzymatic extraction (UAEE), ultrasound-microwave-assisted extraction (UMAE), microwave-assisted extraction (MAE), and high-speed shearing homogenization extraction (HSHE), respectively; Values represent mean ± standard deviation, and superscripts (a–g) differ significantly (*p* < 0.05) among PPPs; Statistical significances were performed by ANOVA and Duncan’s test.

**Table 2:** Molecular weight (*M_w_*), polydispersity (*M_w_/M_n_*), and monosaccharide composition of PPPs obtained by seven extraction methods.

	PPPs Extracted from the Peduncles of *H. dulcis* ^a^						
	PPP-W	PPP-P	PPP-U	PPP-UE	PPP-UM	PPP-M	PPP-HSH
*M_w_* × 10^4^ (Da, error)							
Fraction 1	-	-	36.12 (±0.158%)	27.68 (±0.147%)	29.06 (±0.164%)	-	-
Fraction 2	5.077 (±0.303%)	4.905 (±0.276%)	7.753 (±0.167%)	9.113 (±0.122%)	7.563 (±0.165%)	9.170 (±0.282%)	8.474 (±0.610%)
Fraction 3	0.864 (±1.324%)	0.794 (±0.990%)	1.865 (±0.705%)	1.351 (±0.789%)	1.078 (±0.705%)	1.763 (±0.721%)	1.590 (±0.798%)
*M_w_/M_n_*							
Fraction 1	-	-	1.155 (±0.218%)	1.161 (±0.190%)	1.156 (±0.217%)	-	-
Fraction 2	1.658 (±0.554%)	1.702 (±0.489%)	1.349 (±0.290%)	1.076 (±0.177%)	1.250 (±0.271%)	1.083 (±0.396%)	1.305 (±0.907%)
Fraction 3	1.074 (±1.761%)	1.074 (±1.333%)	1.104 (±0.984%)	1.115 (±1.176%)	1.154 (±0.986%)	1.138 (±1.003%)	1.105 (±1.115%)
Monosaccharide compositions (molar ratio)							
Galacturonic acid	1.00	1.00	1.00	1.00	1.00	1.00	1.00
Galactose	0.93	1.06	1.74	3.43	3.77	2.94	2.16
Arabinose	0.97	1.15	1.54	3.23	3.51	2.72	1.74
Mannose	0.43	0.29	0.42	1.95	1.07	0.69	0.55
Rhamnose	0.48	0.57	0.98	1.80	2.03	1.38	1.14
Glucuronic acid	0.05	0.06	0.09	0.14	0.17	0.16	0.12
Glucose	0.76	1.10	1.84	9.74	6.42	1.90	0.96
Xylose	0.06	0.08	0.07	0.21	0.21	0.14	0.24

**^a^** The codes of samples were the same as in Table 1; Fractions 1–3 were the same as in Figure 1; Values represent mean ± standard deviation; Statistical significances were performed by ANOVA and Duncan’s test.

**Table 3:** Amino acid compositions of PPPs obtained by seven extraction methods.

Amino Acids	PPPs Extracted from the Peduncles of *H. dulcis* ^a^						
	PPP-W (%)	PPP-P (%)	PPP-U (%)	PPP-UE (%)	PPP-UM (%)	PPP-M (%)	PPP-HSH (%)
Aspartic acid	7.01	7.28	10.66	7.96	8.58	8.36	9.25
Threonine	4.83	4.53	7.41	5.61	6.36	6.10	9.26
Serine	6.80	7.23	8.91	7.35	8.14	8.41	8.13
Glutamic acid	13.39	12.98	13.82	14.81	13.45	15.77	9.19
Proline	2.54	3.10	5.45	2.99	3.50	3.06	5.04
Glycine	9.67	10.02	4.42	8.93	4.27	12.10	4.36
Alanine	9.60	9.84	9.47	9.13	9.04	11.20	8.62
Cystine	5.43	5.67	3.90	4.98	5.11	6.55	3.62
Valine	4.07	4.04	4.65	4.45	4.44	3.36	4.06
Isoleucine	7.16	6.57	3.07	7.05	6.69	3.40	6.41
Leucine	7.90	6.99	5.06	7.12	6.93	5.60	6.45
Tyrosine	6.78	7.12	2.40	5.74	5.51	3.38	4.74
Phenylalanine	4.96	4.63	3.23	4.50	4.68	3.92	3.86
Lysine	2.54	2.36	4.99	2.45	3.73	2.10	4.81
Histidine	1.10	0.95	0.78	0.59	0.66	0.79	0.65
Arginine	6.21	6.70	11.76	6.33	8.92	5.89	11.55
Essential amino acids	31.46	29.12	28.41	31.18	32.83	24.48	34.85
Non-essential amino acids	68.54	70.88	71.59	68.82	67.17	75.52	65.15

**^a^** The codes of samples were the same as in Table 1.

**Table 4:** Phenolic compositions of PPPs.

No.	Retention Time (min)	Formula	Molecular Ion [M-H]^−^	Error (ppm)	Score (DB)	Score (MFG)	Identification
1	1.173	C_7_H_6_O_4_	153.0199	3.83	97.75	97.74	Protocatechuic acid ^a,b^
2	1.688	C_15_H_14_O_7_	305.0675	3.48	95.23	95.27	Gallocatechin ^a,b,c^
3	2.188	C_7_H_6_O_3_	137.0247	2.75	85.15	85.19	p-Hydroxybenzoic acid ^a,b^
4	4.185	C_15_H_12_O_8_	319.0467	2.51	97.32	97.27	Ampelopsin ^a,b^
5	7.130	C_27_H_30_O_17_	625.1430	3.00	94.51	94.49	Quercetin-7,4′-diglucoside ^b^
6	7.729	C_15_H_12_O_7_	303.0522	3.76	94.51	94.60	Dihydroquercetin ^b^
7	8.345	C_27_H_30_O_16_	609.1479	2.95	93.82	93.93	Rutin ^a,b,c^
8	8.645	C_21_H_20_O_12_	463.0894	2.46	97.09	97.06	Myricitrin ^b^
9	10.575	C_15_H_10_O_8_	317.0311	2.74	96.81	96.76	Myricetin ^a,b,c^
10	12.239	C_15_H_10_O_7_	301.0363	2.98	96.90	96.87	Quercetin ^a,b,c^
11	12.256	C_15_H_10_O_6_	285.0409	1.45	98.98	98.99	Kaempferol ^a,b,c^
12	12.306	C_16_H_12_O_8_	331.0466	1.99	97.94	97.90	5-Methylmyricetin ^b^
13	12.888	C_15_H_12_O_5_	271.0618	3.16	91.03	91.03	Naringenin ^a,b^

**^a^** Compared with literatures; **^b^** Compared with Traditional Chinese Medicine (TCM)-database; **^c^** Compared with an authentic standard.
